# The unexpected co-occurrence of *GRN* and *MAPT* p.A152T in Basque families: Clinical and pathological characteristics

**DOI:** 10.1371/journal.pone.0178093

**Published:** 2017-06-08

**Authors:** Fermin Moreno, Begoña Indakoetxea, Myriam Barandiaran, María Cristina Caballero, Ana Gorostidi, Francesc Calafell, Alazne Gabilondo, Mikel Tainta, Miren Zulaica, José F. Martí Massó, Adolfo López de Munain, Pascual Sánchez-Juan, Suzee E. Lee

**Affiliations:** 1 Department of Neurology, Hospital Universitario Donostia, San Sebastian, Spain; 2 Centro de Investigación Biomédica en Red sobre Enfermedades Neurodegenerativas (CIBERNED), Institute Carlos III, Madrid, Spain; 3 Neuroscience Area, Institute Biodonostia, San Sebastian, Spain; 4 Department of Pathology, Hospital Universitario Donostia, San Sebastián, Spain; 5 Brain Bank Hospital Universitario Donostia, from the Basque Biobank for Research (OEHUN), San Sebastian, Spain; 6 Institute of Evolutionary Biology (CSIC-UPF), Department of Experimental and Health Sciences, Universitat Pompeu Fabra, Barcelona, Spain; 7 Department of Neurology, Hospital de Bidasoa, Irun, Spain; 8 Department of Neurosciences, Universidad del País Vasco UPV-EHU, San Sebastian, Spain; 9 Neurology Service and Centro de Investigación Biomédica en Red sobre Enfermedades Neurodegenerativas (CIBERNED), ‘Marqués de Valdecilla’ University Hospital, University of Cantabria, Institute for Research ‘Marqués de Valdecilla’ (IDIVAL), Santander, Spain; 10 Department of Neurology, Memory and Aging Center, University of California San Francisco, San Francisco, United States of America; Institut Pasteur de Lille, FRANCE

## Abstract

**Background:**

The co-occurrence of the c.709-1G>A *GRN* mutation and the p.A152T *MAPT* variant has been identified in 18 Basque families affected by frontotemporal dementia (FTD). We aimed to investigate the influence of the p.A152T *MAPT* variant on the clinical and neuropathological features of these Basque *GRN* families.

**Methods and findings:**

We compared clinical characteristics of 14 patients who carried the c.709-1G>A *GRN* mutation (*GRN*+/A152T-) with 21 patients who carried both the c.709-1G>A *GRN* mutation and the p.A152T *MAPT* variant (*GRN*+/A152T+). Neuropsychological data (n = 17) and plasma progranulin levels (n = 23) were compared between groups, and 7 subjects underwent neuropathological studies. We genotyped six short tandem repeat markers in the two largest families. By the analysis of linkage disequilibrium decay in the haplotype block we estimated the time when the first ancestor to carry both genetic variants emerged.

*GRN*+/A152T+ and *GRN*+/A152T- patients shared similar clinical and neuropsychological features and plasma progranulin levels. All were diagnosed with an FTD disorder, including behavioral variant FTD or non fluent / agrammatic variant primary progressive aphasia, and shared a similar pattern of neuropsychological deficits, predominantly in executive function, memory, and language. All seven participants with available brain autopsies (6 *GRN*+/A152T+, 1 *GRN*+/A152T-) showed frontotemporal lobar degeneration with TDP-43 inclusions (type A classification), which is characteristic of *GRN* carriers. Additionally, all seven showed mild to moderate tau inclusion burden: five cases lacked β-amyloid pathology and two cases had Alzheimer’s pathology. The co-occurrence of both genes within one individual is recent, with the birth of the first *GRN*+/A152T+ individual estimated to be within the last 50 generations (95% probability).

**Conclusions:**

In our sample, the p.A152T *MAPT* variant does not appear to show a discernible influence on the clinical phenotype of *GRN* carriers. Whether p.A152T confers a greater than expected propensity for tau pathology in these *GRN* carriers remains an open question.

## Introduction

In 2008, our group described a cluster of families with frontotemporal dementia (FTD) harboring the c.709-1G>A mutation in the progranulin gene (*GRN)*, a mutation unique to individuals in the Basque country [1]. The most common presenting clinical syndromes of these Basque *GRN* carriers include behavioral variant FTD (bvFTD), followed by nonfluent variant primary progressive aphasia (nfvPPA), with about half of these patients developing features of corticobasal syndrome (CBS) throughout their disease course [2]. The clinical phenotype of the Basque *GRN* carriers is variable even within families, as has been reported for other *GRN* mutations [3–5]. More recently, we found that 71% of these Basque *GRN* carriers also carry a rare variant in the microtubule-associated protein tau gene (*MAPT*) in exon 7 (p.A152T) [6], which has been linked to risk for both FTD-spectrum disorders and clinical Alzheimer’s disease (AD) [7,8]. The frequency of the *MAPT* p.A152T variant is very low in healthy controls (0.25–0.71%) but is overrepresented in patients with FTD (0.72–1.42%) and AD (0.56–1.02%) [6,7]. Although the p.A152T residue lies outside of the microtubule-binding region on tau, functional studies suggest that the p.A152T variant leads to tauopathy and axonal degeneration [7,9,10].

The co-occurrence of *GRN*, a pathogenic frontotemporal lobar degeneration (FTLD) mutation, and p.A152T, a genetic risk factor for FTD and AD, in a well-characterized group of patients enables us to determine the influence of these genes on disease phenotype. Among these Basque families, *MAPT* p.A152T has been found to either completely or partially co-segregate with *GRN* c.709-1G>A [6], which may be due to the fact that the two genes, *GRN* and *MAPT*, are located only 1.7 Mb from each other on chromosome 17. Overall, *MAPT* p.A152T was present in nine of the fifteen families [6]. The aim of this study was to explore and date the origin of these genes in the Basque families and to compare the clinical and neuropathological phenotypes of *GRN* carriers with and without p.A152T.

## Methods

### Participants

Thirty-five patients who carried the c.709-1G>A *GRN* mutation were included in the clinical analysis. Clinical diagnoses were reviewed in a consensus conference with three experienced neurologists (FM, BI, and ALdM) and a neuropsychologist (MB) using clinical criteria for bvFTD [11], primary progressive aphasia [12] and CBS [13]. We also assessed age of symptom onset and defined motor onset as the time from symptom onset to the development of motor symptoms. Motor symptoms included gait impairment, falls, dysequilibrium, weakness or adventitious movements. Motor signs on neurological examination included rigidity, tremor, bradykinesia, dysequilibrium, or gait disturbance. For the 21 patients with available structural MRI, the presence or absence of asymmetry was evaluated based on visual inspection.

To estimate the frequency of both genes in the Basque families, we analyzed DNA of 102 subjects, which included the 35 symptomatic patients mentioned above and 67 asymptomatic members of these c.709-1G>A *GRN* families. To analyze the prevalence of the p.A152T variant in the Basque region, we also screened 507 unrelated healthy controls. Healthy controls were required to have no known neurological disease or cognitive complaints. Moreover, 57 patients with Parkinson’s disease carrying the R1441 or G2019S mutations in the leucine-rich repeat kinase 2 gene (*LRRK2*) and 20 patients with myotonic dystrophy type 1 (Steinert disease) were screened for p.A152T. This study was approved by the Donostia Hospital Ethics Committee and written informed consent was obtained from all participants or their surrogates.

### Neuropsychological assessment

Eighteen patients were evaluated with a standardized neuropsychological assessment within the first year of symptom onset. Overall cognitive status was assessed using the Mini-Mental State Examination (MMSE) [14]. Verbal episodic memory and design copy were tested using the Word List and the Constructional Praxis subtests from the Consortium to Establish a Registry for Alzheimer’s Disease (CERAD) [15]. The battery also included the Trail Making Test Parts A and B (TMT-A and–B) [16] and the GERMCIDE neuropsychological protocol [17] that is comprised of digit span forward and backward, semantic (animals) and phonetic verbal fluency, verbal comprehension and repetition, reasoning and unilateral and bilateral praxis tests. Twelve patients (6 *GRN*+/A152T+ and 6 *GRN*+/A152T- underwent follow up neuropsychological testing an average of 12.3± 0.8 months after the first assessment. Change scores in MMSE between baseline and follow up were compared between the two groups using a two sample t-test.

### Progranulin levels

We compared available plasma progranulin (PGRN) levels of 23 patients, which included 9 *GRN*+/A152T+ and 14 *GRN*+/A152T- carriers. Combining all 23 patients into one group, we correlated PGRN levels with age of onset and also compared PGRN levels between clinical syndromes. Plasma samples were diluted 1:100 in the dilution buffer provided and levels of PGRN were measured in a duplicate set using a commercial ELISA assay (Progranulin [human] ELISA kit, AdipoGen, Inc, South Korea; ref AG-45A-0018YEK-KI01) according to the manufacturer’s instructions. Recombinant human PGRN provided with the ELISA kit was used as a standard.

### Genetic studies

Whole blood DNA was extracted using standard procedures. *MAPT* SNP rs143624519 genotyping was performed with a TaqMan allelic discrimination assay with inventoried probes (Applied Biosystems) and a LightCycler96 PCR system. Genotyping calls were made using LightCycler96 SW1.1 software (Roche).

To analyze the haplotypes and estimate the age of the mutation, we decided to base the analysis on the two largest families, Family 1 and Family 2, for which we had DNA samples from 21 and 26 individuals respectively. To examine the haplotype surrounding *GRN* mutation c.709-1G>A and *MAPT* SNP rs143624519, we typed six short tandem repeat (STR) markers spanning a region of 4.46 Mb on chromosome 17q21. All markers (D17S930, D17S1861, D17S950, D17S934, D17S920, and D17S1868) were amplified with one fluorescently labeled primer. PCR fragments were analyzed on an ABI 3100 automated DNA analyzer and alleles were scored using the GeneMapper software (both from Applied Biosystems).

Haplotypes were resolved with PHASE 2.1 [18,19]. The age of the *GRN* mutation c.709-1G>A was estimated from Equation 28 in Walsh et al. [20], which estimates the time to the most recent common ancestor of two individuals based on their genetic differences. We set a general STR mutation rate of m = 0.001, together with an estimated recombination rate between the extreme markers in the haplotype of r = 0.022 (from Kong et al. [21]).

### Neuropathological studies

Neuropathological studies were carried out at Donostia University Hospital, using samples from the Neurological Tissue Bank of the Basque Biobank for Research (OEHUN), according to standardized protocols. The right half of the brain was sliced fresh and stored frozen at -80°C. The left half of the brain was fixed in 10% buffered formaldehyde for 3 to 4 weeks. The histological assessment was performed in 5-μm-thick sections of formalin-fixed paraffin-embedded blocks of tissue from areas of the left brain, namely, the frontal, temporal, parietal and occipital cortex, motor cortex, anterior and posterior cingulate cortices, basal ganglia (including striatum and globus pallidus), nucleus basalis of Meynert, thalamus, amygdala, anterior and posterior hippocampus with parahippocampal gyrus, midbrain (with rostral and caudal substantia nigra), pons with locus coeruleus, medulla oblongata, cerebellar vermis, dentate nucleus of the cerebellum, and olfactory bulb.

Sections of each tissue block were stained with hematoxylin and eosin; additionally, Luxol fast blue counterstained with periodic acid-Schiff (PAS) was used on certain blocks. Automated immunohistochemistry was performed using primary antibodies on a Ventana BenchMark ULTRA slide staining system (Ventana Medical Systems, Tucson, USA). The Ventana staining procedure included pretreatment with cell conditioner 1 (pH 8) or 2 (pH 6) and formic acid, depending on the antibody, followed by incubation with the corresponding antibody. This incubation was followed by treatment with ultraView Universal DAB. The primary antibodies used were: anti-β-amyloid (dilution 1:50, monoclonal, clone 12F4, Covance), anti-PHF tau (dilution 1/2000, monoclonal, clone AT8, Innogenetics), anti-α-synuclein (dilution 1:4000, monoclonal, clone LB509, Covance), anti-TDP-43 (dilution 1:1000, monoclonal, clone 2E2-D3, Abnova), anti-ubiquitin (dilution 1:300, polyclonal, Dako), anti-PrP (dilution 1:100, monoclonal, clone 3F4, Millipore), anti-tau (4-repeat isoform RD4) (dilution 1:80, monoclonal, clone 1E1/A6, Millipore), anti-tau (3-repeat isoform RD3) (dilution 1:300, monoclonal, clone 8E6/C11, Millipore), neurofilament (pre-diluted, monoclonal, clone 2F11, Roche), and anti-α B-crystallin (dilution 1:500, polyclonal, ABN185, Millipore).

The semi-quantitative analysis of tau inclusions was performed by a neuropathologist blinded to the genetic status of the patients according to this scheme: -: no inclusions; +: mild density (isolated tau inclusions, barely noted at low magnification (x100); ++: moderate density (moderate tau inclusions, easily visible at low magnification (x100); +++: high density (abundant tau inclusions).

### Statistical analysis

For comparisons between groups, categorical variables were compared with the χ^2^ test or the Fisher exact test as appropriate. Continuous variables were compared with a t-test or the Mann-Whitney U test as appropriate for the data, and with a one-way analysis of variance (ANOVA) when more than two groups were included in the analysis. The Pearson correlation coefficient was used in order to study the linear association between continuous variables, and the Shapiro-Wilk test determined normality of variables for each group considered. The Levene’s test was used to check equality between the variances. Statistical analysis was performed using IBM SPSS Statistics for Windows, Version 17.0.0.

## Results

Since our previous study revealed that 71% of c.709-1G>A *GRN* mutation carriers also carried the p.A152T variant [6], we screened additional Basque individuals in present study to determine whether the variant occurs more frequently in the Basque population. This screen included 507 healthy controls and patients with genetic neurological diseases that have a higher prevalence in the Basque population, including 20 patients with myotonic dystrophy type 1 and 57 with Parkinson’s disease related to the *LRRK2* mutation [22]. We found seven p.A152T healthy control carriers, reflecting a carrier rate of 1.4%. There was one p.A152T carrier among 57 *LRRK2* carriers and no p.A152T carriers were identified among 20 patients with myotonic dystrophy type 1. Among the 102 individuals in these *GRN* families with available DNA, we found that 37 out of 60 *GRN* mutation carriers also had the p.A152T variant (61.7% overall, 60% of affected carriers and 64% of presymptomatic carriers) and 5 out of 42 *GRN* negative family members (11.9%) carried the p.A152T variant (*GRN*-/A152T+, Table 1). At the time of this study, none of the five *GRN*-/A152T+ had developed neurodegenerative disease, cognitive complaints or behavioral changes. Among the five *GRN*-/A152T+, three were in their eighties, one was in his fifties and one was in her early forties. Linkage disequilibrium analysis revealed that *GRN* and p.A152T (located on chromosome 17) were in partial linkage disequilibrium (D' = 0.78; r^2 =^ 0.46).

**Table 1:** *MAPT* p.A152T and *GRN* mutation frequencies in 102 Basque *GRN* family members.

	Symptomatic (n)	Asymptomatic (n)	Total (n)
***GRN*+/A152T+**	21	16	37
***GRN*+/A152T-**	14	9	23
***GRN*-/A152T+**	0	5	5
***GRN*-/A152T-**	0	37	37
**Total**	35	67	102

*GRN*+: *GRN* c.709-1G>A mutation carrier; *GRN*-: non-carrier of the *GRN* c.709-1G>A mutation; A152T+: *MAPT* p.A152T carrier; A152T-: non-carrier of *MAPT* p.A152T.

### Origin of the mutation

Short Tandem Repeat (STR) haplotype data were available for 5 *GRN*+ and 16 *GRN*- individuals in Family 1 and for 12 *GRN*+ and 14 *GRN*- individuals in Family 2. Both families carried the *GRN* c.709-1G>A mutation in the same haplotype, in alleles 106, 91, 128, 185, A, 98 and 185 at the following loci: D17S930, D17S1861, D17S934, D17S950, rs14362519, D17S920 and D17S1868, respectively. Note that nucleotide A at rs143624519 corresponds to the T amino acid substitution at *MAPT* A152T. This haplotype was not found in a sample of 18 control chromosomes (which all carried various different haplotypes) nor was it found in the 77 *GRN*- chromosomes from the affected families. Hence, these two families share the *GRN* c.709-1G>A mutation by common descent and the mutation did not arise in each family as two independent events. Using Equation 28 from Walsh et al. [20], the mutation was estimated with 95% probability to have arisen 0 to 49 generations ago.

In Family 2, three *GRN*+/A152T- siblings were identified. However, their STR haplotypes were consistent with a single recombination event between D17S950 and A152T in the transmission of their *GRN* mutation from their maternal grandfather to their mother, as can be reconstructed from other descendants of their maternal grandfather (see supporting information for pedigrees).

### Clinical phenotype

We compared 21 *GRN*+/A152T+ and 14 *GRN*+/A152T- patients for differences in the following clinical variables: age of onset, initial clinical diagnosis, time to development of motor symptoms, the development of a secondary symptom of CBS, and asymmetry on structural MRI. For both *GRN*+/A152T+ and *GRN*+/A152T-, the most common clinical syndrome was bvFTD (48% and 64%), followed by nfvPPA (24% and 14%), while AD and CBS were less common presenting syndromes (Table 2). Patients diagnosed with bvFTD (10 in the GRN+/A152T+ group and 9 in the GRN+/A152T- group) presented primarily with apathy, decreased spontaneous speech output, deficits in planning and organizing, memory complaints, neglect of self-care and increased appetite, with no apparent difference between genetic groups. All patients diagnosed with nfvPPA (5 in the *GRN*+/A152T+ group and 2 in the *GRN*+/A152T- group) presented with anomia with non-fluent, effortful speech of varying degree, with diminished spontaneous speech output, articulation deficits, phonemic paraphasias, echolalia and dysgraphia. Most nfvPPA also featured some degree of impaired sentence comprehension. Overall, language features in patients with nfvPPA appeared similar in both genetic groups. Patients diagnosed with AD (3 *GRN*+/A152T+ and 2 GRN+/A152T-) had an amnestic syndrome with prominent short-term memory deficits and no apparent behavioral or motor symptoms. One *GRN*+/A152T+ patient with CBS had asymmetric rigidity and bradykinesia associated with limb apraxia.

**Table 2:** Comparison of demographic and clinical features between groups.

	21 GRN+/A152T+	14 GRN+/A152T-	*p*
**Sex (female %)**	71.4%	50%	0.29
**Age of onset (years)**	61.0 ± 7.1	61.4 ± 9.2	0.62
**Initial diagnosis**	bvFTD (10)	bvFTD (9)	0.98
	nfvPPA (5)	nfvPPA (2)	
	AD (3)	AD (2)	
	CBS (1)	CBS (0)	
	Dementia NOS (2)	PD (1)	
**Time to onset of motor symptoms (years)**	3.1 ± 1.3	3.1 ± 1.7	0.84
**% developing CBS throughout the disease course**	57.1%	35.7%	0.31
**% with asymmetry on structural MRI**	54.5%	70%	0.66

AD: Alzheimer’s disease; bvFTD: behavioral variant frontotemporal dementia; CBS: corticobasal syndrome; nfvPPA: nonfluent/agrammatic variant of primary progressive aphasia. NOS: not otherwise specified.

Two *GRN*+/A152T+ and one *GRN*+/A152T- had syndromes that were not consistent with the four main syndromes in this cohort. These included two *GRN*+/A152T+ patients with dementia affecting multiple cognitive domains. The first patient had memory impairment, anomia, and ideomotor apraxia without evident behavioral symptoms. The second patient presented with memory impairment, anomia and comprehension impairment, and depressive symptoms. Neither of these two patients met bvFTD or nfvPPA criteria. One *GRN*+/A152T- patient was diagnosed with clinical Parkinson’s disease (PD) at first presentation because his first symptoms included left-sided rest tremor, rigidity and bradykinesia, and a gait abnormality featuring truncal anteflexion and diminished arm swing. He did not improve on levodopa. He later developed left ideomotor apraxia, focal reflex myoclonus and cortical sensory loss consistent with a diagnosis of corticobasal syndrome.

Although each patient’s behavior changes were not formally assessed with behavioral scales, apathy, impulsivity and increased appetite represented the most common behavioral symptoms. Agitation, hallucinations, delusions and euphoria emerged infrequently, with each present in less than 15% of subjects.

The *GRN*+/A152T+ group showed a higher percentage of subjects developing CBS over the course of the disease (57.1% vs. 35.7%) compared to *GRN*+/A152T-, although the difference was non-significant. *GRN*+/A152T+ and *GRN*+/A152T- showed similar mean age of symptom onset, about 61 years. Time from symptom onset to the development of motor symptoms was also similar in the two groups. The majority of patients had asymmetrical atrophy on visual inspection, and there were no significant differences in brain asymmetry between groups.

### Neuropsychological phenotype

We compared neuropsychological performance of 8 *GRN*+/A152T+ with 9 *GRN*+/A152T- and found no significant differences between groups for any individual tests. Overall, both groups showed impairments in executive function, memory, and language. The *GRN*+/A152T+ patients had lower scores in measures of executive function, including trail making A and B and semantic and phonemic verbal fluency, and working memory (digits backward), although the results did not reach statistical significance. Both groups also showed similar performance on tests of attention (digit span forward), memory (verbal episodic memory with the Word List from the CERAD) and praxis. When comparing changes in the MMSE score for 6 *GRN*+/A152T+ vs. 6 *GRN*+/A152T-, we found that the *GRN*+/A152T+ group had a non-significant trend for greater decline in MMSE score over 1 year (Table 3).

**Table 3:** Comparison of neuropsychological performance between groups.

	8 *GRN+/A152T+*	9 *GRN+/A152T-*	*p*
***MMSE***	23.6 ± 4.4	24.5 ± 3.9	0.66
***Digit span forward***	4.7 ± 0.7	4.6 ± 0.7	0.66
***Digit span backward***	2.4 ± 1.3	3.1 ± 0.7	0.15
***TMT-A***	100.8 ± 50.6	76.8 ± 26.3	0.34
***TMT-B***	200 ± 36.7	180.2 ± 73.9	0.60
***Semantic verbal fluency***	9.9 ± 4.0	11.3 ± 4.5	0.50
***Phonetic verbal fluency***	3.7 ± 3.1	7.3 ± 6.2	0.23
***Reasoning***	5.1 ± 3.5	4.3 ± 2.1	0.54
***Verbal comprehension***	5.6 ± 0.7	5.9 ± 0.3	0.35
***Repetition***	9.9 ± 0.3	9.9 ± 0.3	0.93
***Unilateral praxis***	8.6 ± 3.2	9.8 ± 0.4	0.34
***Bilateral praxis***	6.1 ± 2.3	6.4 ± 1.7	0.75
***CERAD Word List 1***	2.2 ± 1.4	2.7 ± 0.9	0.43
***CERAD Word List 2***	3.6 ± 1.5	4.1 ± 1.1	0.45
***CERAD Word List 3***	4.7 ± 2.0	5.2 ± 2.1	0.65
***CERAD Word List Recall***	3.2 ± 1.5	2.4± 2.0	0.34
***CERAD Word List Recognition***	17.5 ± 1.9	16.3 ± 3.6	0.38
***CERAD Constructional Praxis***	9.1 ± 3.2	9.3 ± 1.8	0.89
***MMSE score change at 1 year***	-5.2 ± 3.8	-3.7 ± 2.8	0.45

CERAD: Consortium to Establish a Registry for Alzheimer’s Disease; MMSE: Mini-Mental State Examination; TMT: Trail Making Test.

### Progranulin levels

*GRN*+/A152T+ showed lower mean progranulin levels (mean: 54 ± 19.1 ng/ml) compared with those carrying only the *GRN* mutation (mean: 72.5 ± 31.4 ng/ml), although these results did not reach statistical significance (Mann-Whitney’s U test: p = 0.201). Interestingly, considering the distribution of serum PGRN levels, we observed that 5 out of 14 patients (35.7%) in the *GRN*+/A152T+ had levels of PGRN below 45 ng/ml and 11 out of 14 (78.6%) below 70 ng/ml, while these percentages were 0% and 55.5% respectively in the group carrying only the *GRN* mutation. Across all patients, PGRN levels did not significantly correlate with the age of onset (Pearson correlation coefficient, r = -0.260, p = 0.268). There were no differences in PGRN levels when patients with different clinical syndromes (bvFTD, PNFA, AD, dementia not otherwise specified and PD) were compared (ANOVA, F = 0.145, p = 0.706). Because of small sample sizes, we did not perform subgroup analyses comparing the relationship of PGRN levels versus age of onset between *GRN*+/A152T+ and *GRN*+/A152T- or plasma PGRN stratified by clinical and genetic subtype.

### Neuropathological studies

**Fig 1:**
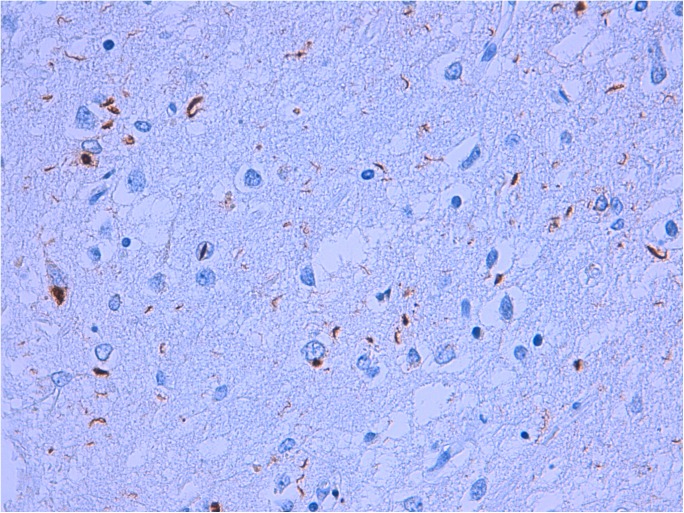
Frontotemporal lobar degeneration with TDP-43 type A from a GRN+/A152T+ patient. Numerous neuronal cytoplasmic inclusions and dystrophic neurites with occasional neuronal intranuclear inclusions in the superficial cortex.

We performed neuropathological assessment in seven patients, including six *GRN*+/A152T+ and one *GRN*+/A152T-. All seven showed histopathological features of FTLD with TAR DNA-binding protein 43 (TDP-43)-positive inclusions (FTLD-TDP) type A (Fig 1), which is characteristic of *GRN* mutation carriers [23,24]. Two of the patients who carried both the *GRN* mutation and p.A152T also had Alzheimer’s disease co-pathology. One patient showed a low density of neuropil threads and pretangles in transentorhinal cortex, β-amyloid deposits in hippocampus, amygdala, nucleus basalis of Meynert, striatum, insular and cingulate cortex and neocortex. β-amyloid pathology was absent in midbrain and cerebellum. There were isolated neuritic plaques in neocortex. These findings correspond to a diagnosis of AD neuropathologic change: A2B1C1, according to the National Institute on Aging-Alzheimer’s Association (NIA-AA) guidelines for the neuropathologic assessment of AD [25]. The second patient with AD co-pathology showed a high density of neuropil threads and neurofibrillary tangles in entorhinal and transentorhinal cortices, and a moderate density of neurofibrillary tangles in hippocampus and middle temporal gyrus. β-amyloid deposits were present in neocortex, hippocampus, amygdala, nucleus basalis of Meynert, cingulate cortex, neocortex, substantia nigra and cerebellum. There were also β-amyloid deposits in meningeal vessels. Isolated neuritic plaques were present in temporal neocortex. These findings correspond to a diagnosis of Alzheimer disease neuropathologic change: A3B2C1, according to the NIA-AA guidelines [25].

**Fig 2:**
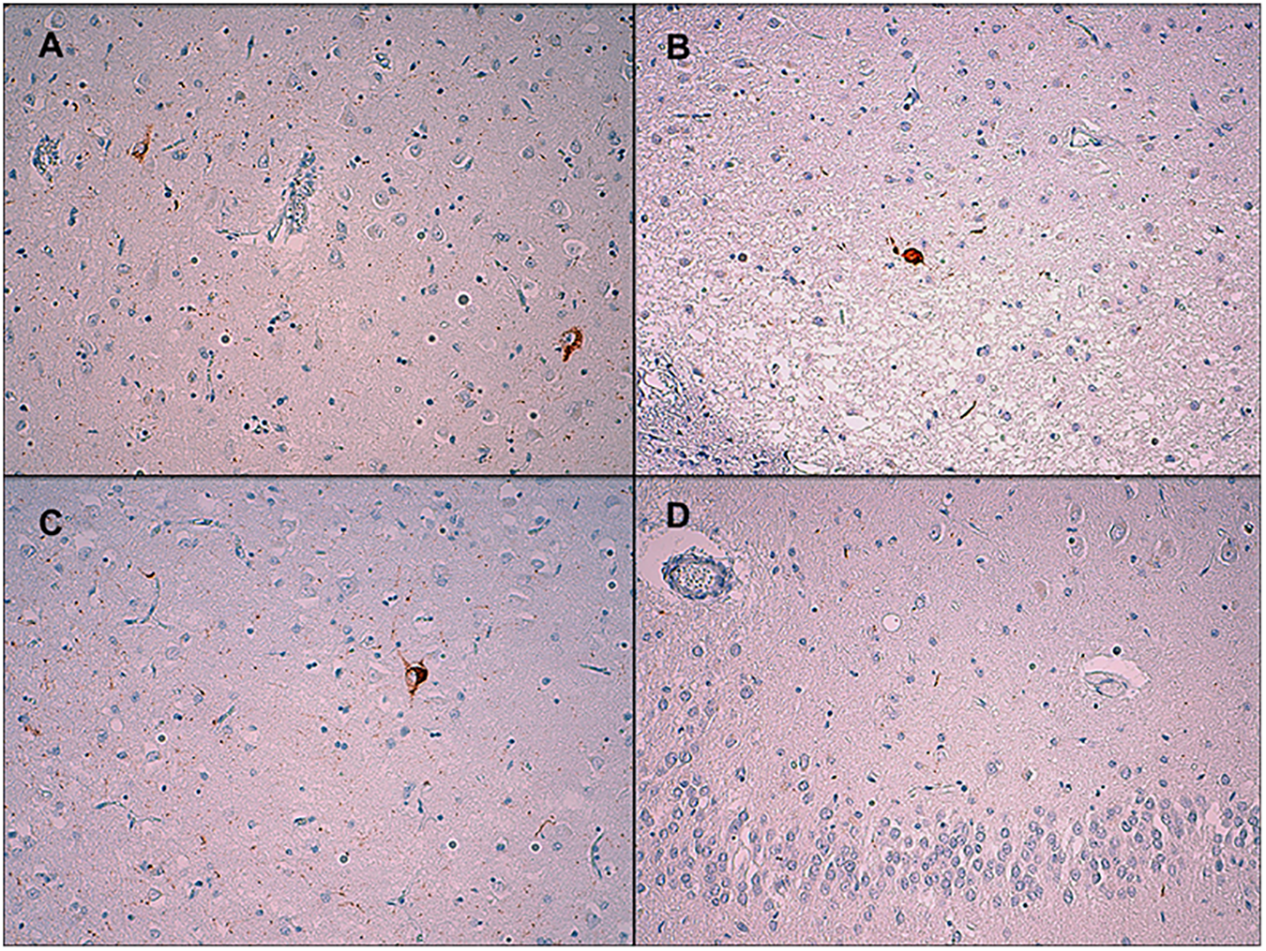
Tau immunohistochemistry from a *GRN*+/A152T+ patient. (A) Mild immunoreactivity in the neuronal cytoplasm and threads in the cerebral cortex, x200. (B) Isolated immunoreactivity in the neuronal cytoplasm and processes in the basal ganglia, x200. (C) Mild immunoreactivity in the cytoplasm and processes in the amygdala, x200. (D) Mild immunoreactivity in neuronal processes and lack of immunoreactivity in neuronal cytoplasm in the dentate gyrus of the hippocampus, x200.

**Fig 3:**
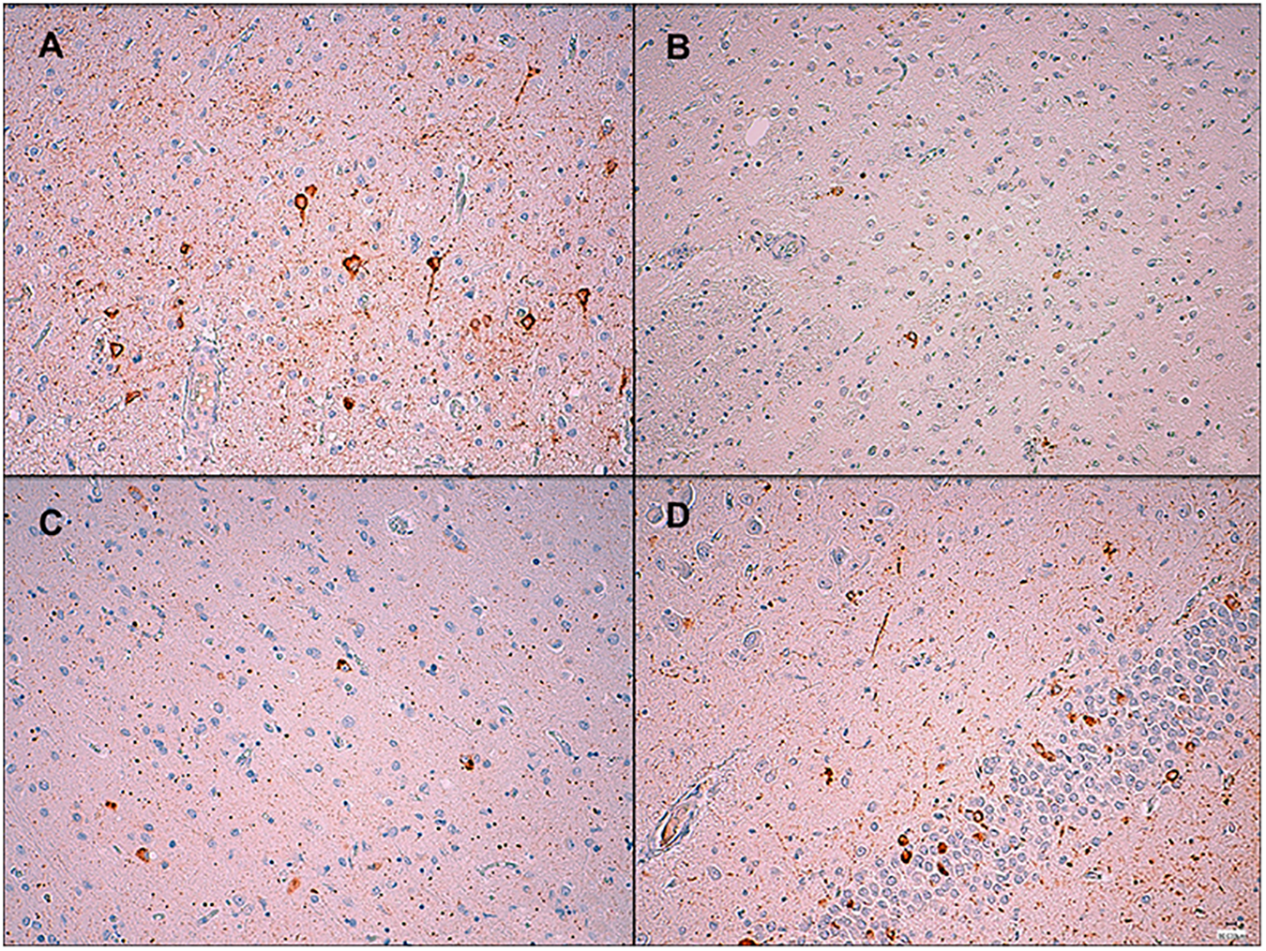
Tau immunohistochemistry from a *GRN*+/A152T+ patient diagnosed with unclassifiable tauopathy. (A) Strong diffuse neuronal cytoplasm immunoreactivity and threads in the cerebral cortex, x200. (B) Mild immunoreactivity in the neuronal cytoplasm and processes in the basal ganglia, x200. (C) Moderate diffuse neuronal cytoplasm immunoreactivity, some neurofibrillary tangles and grains in the neuronal processes in the amygdala, x200. (D) Moderate diffuse neuronal cytoplasm immunoreactivity in the neuronal cytoplasm of the dentate gyrus and threads in the hippocampus, x200.

When we found that the p.A152T variant showed a high frequency in these *GRN* families, a neuropathologist (MCC), blinded to the individuals’ genetic status, performed a semi-quantitative analysis of tau pathology in these brains. All of the autopsies showed mild to moderate tau pathology (Table 4). The tau inclusions seen included neuropil threads (NTs), neurofibrillary tangles (NFTs), pretangles (PTs) and astrocytic inclusions, with NTs and PTs showing a higher burden across the cases (Figs 2 and 3). The amygdala, entorhinal and transentorhinal cortex and nucleus basalis of Meynert consistently showed tau pathology. The striatum was usually more affected than the globus pallidus, and in the neocortex, the tau pathology was most prominent in the temporal lobe, followed by the frontal and occipital lobes. In most cases, except in the two with a diagnosis of Alzheimer’s disease, these tau inclusions were not accompanied by β-amyloid pathology. One patient from the *GRN*+/A152T+ group received an additional diagnosis of unclassifiable tauopathy because in addition to FTLD-TDP A, tau burden was unusually diffuse and severe, with tau immunoreactivity found in astrocytes in a distribution that did not clearly fit a defined tauopathy. We found abundant tau-positive deposits in the cortex in neuronal cytoplasm and threads and grains in the neuropil. There were some coiled bodies and astrocytic deposits. These inclusions were more abundant in temporal neocortex than in frontal and parietal cortex. In the striatum, there were moderate tau immunoreactive deposits in neuronal cytoplasm and neuropil. In the hippocampus, we found neuronal cytoplasmic inclusions in the dentate gyrus and CA1, C2, C3 and C4 neurons, but also some threads, grains, and coiled bodies in the white matter. Abundant tau positive inclusions were seen in the entorhinal and transentorhinal cortex. We found also tau positive inclusions in the subthalamic nucleus, substantia nigra and locus coeruleus. There were no tufted astrocytes, astrocytic plaques or Pick bodies. Immunohistochemistry to determine tau isoforms was performed with a positive control in every slide and revealed a mixed 3R and 4R tauopathy. We found immunoreactive 3R/4R inclusions in the nucleus basalis of Meynert (neurofibrillary tangles, isolated granular deposits), amygdala (sparse cytoplasmic neuronal inclusions), and anterior hippocampus (granular immunoreactive deposits in neuronal cytoplasm). Isolated 4R isoform was also present in amygdala (neuropil), substantia nigra, anterior hippocampus (grains in the neuropil) and in oligodendroglia. The immunohistochemistry for tau isoforms in the neocortex was inconclusive (Fig 3).

**Table 4:** Semi-quantitative analysis of tau pathology in individual autopsies.

	Tau pathology	Frontal cortex	Temporal cortex	Occipital cortex	Striatum	Globus pallidus	Thalamus	Nucleus basalis of Meynert	Amygdala	Entorhinal cortex	Substantia nigra	Locus coeruleus	Olfactory bulb
**FTLD-TDP type A (57 years, GRN+/p.A152T-)**	NTs	++	+	+	+	-	-	+	+	+	+	-	+
	PTs	+	+	-	-	-	+	+	+	+	-	-	+
	NFTs	+	-	-	-	-	-	-	+	-	-	-	-
	NPs	-	-	-	-	-	-	-	-	-	-	-	-
	ASTs	-	-	-	-	-	-	-	-	-	-	-	-
**FTLD-TDP type A (64 years, GRN+/p.A152T+)**	NTs	+	+	+	+	-	-	+	+	+	+	+	+
	PTs	+	+	-	-	-	-	-	+	++	-	-	-
	NFTs	-	-	-	-	-	-	-	-	-	-	-	-
	NPs	-	-	-	-	-	-	-	-	-	-	-	-
	ASTs	-	-	-	-	-	-	-	-	-	-	-	-
**FTLD-TDP type A(70 years, GRN+/p.A152T+)**	NTs	-	+	-	-	+	-	+	+	+	+	+	-
	PTs	-	-	-	-	+	-	-	+	+	-	+	-
	NFTs	-	-	-	-	-	-	-	+	-	+	-	-
	NPs	-	-	-	-	-	-	-	-	-	-	-	-
	ASTs	-	-	-	-	-	-	-	-	-	-	-	-
**FTLD-TDP type A (76 years, GRN+/p.A152T+)**	NTs	-	+	-	-	-	-	+	+	+	-	-	-
	PTs	-	+	-	-	-	-	+	+	+	-	-	-
	NFTs	-	+	-	-	-	-	+	+	-	-	-	-
	NPs	-	-	-	-	-	-	-	-	-	-	-	-
	ASTs	-	-	-	-	-	-	-	-	-	-	-	-
**FTLD-TDP type A + unclassifiable tauopathy(75 years, GRN+/p.A152T+)**	NTs	+	+++	+	+	++	+	+++	+++	+++	++	++	+
	PTs	+	+++	-	++	++	-	+++	+++	+++	-	++	+
	NFTs	-	+++	+	++	+	+	+	++	+	++	+	-
	NPs	-	-	-	-	-	-	-	-	-	-	-	-
	ASTs	+	++	+	+	-	-	-	++	+	-	-	-
**FTLD-TDP type A + Alzheimer’s disease(71 years, GRN+/p.A152T+))**	NTs	+	++	-	+	-	-	+	-	+	+	++	-
	PTs	-	+	-	+	-	+	+	++	+	-	+++	-
	NFTs	-	+	-	-	-	-	-	+	-	-	+	-
	NPs	-	+	-	-	-	-	-	-	-	-	-	-
	ASTs	-	-	-	-	-	-	-	-	-	-	-	-
**FTLD-TDP type A + Alzheimer’s disease(76 years, GRN+/p.A152T+)**	NTs	+	+++	+	+	+	+	+	+++	+++	+	++	+
	PTS	+	-	+	+	-	-	+	+	+	+	+	+
	NFTs	+	++	-	-	-	+	++	++	+++	-	+	-
	NPSs	-	+	-	-	-	-	-	+++	+++	-	-	-
	ASTs	-	-	-	-	-	-	-	-	-	-	-	-

ASTs: tau-positive astrocytic inclusions; NFTs: neurofibrillary tangles; NPs: neuritic plaques; NTs: neuropil threads; PTs: pretangles. -: no inclusions; +: mild density (isolated tau inclusions, barely noted at low magnification (x100); ++: moderate density (moderate tau inclusions, easily visible at low magnification (x100); +++: high density (abundant tau inclusions).

## Discussion

Here, we describe the clinical and pathological features of a cluster of families that share the private c.709-1G>A *GRN* mutation for FTLD and the p.A152T *MAPT* variant, which has been identified as a risk factor for AD and FTD-spectrum disorders. Across these 18 Basque families, we have identified 37 individuals with both genes, 23 who carry only the c.709-1G>A *GRN* mutation, and five who carry only the p.A152T *MAPT* variant. Those carrying only the *MAPT* variant have not developed clinical symptoms. In the families for which we have STR genotypes, it is clear that the c.709-1G>A *GRN* mutation resulted from a single event, in a haplotype background carrying p.A152T. For some individuals, the p.A152T *MAPT* variant has been lost due to recombination. The c.709-1G>A *GRN* mutation is estimated to have arisen within the last 50 generations, that is, roughly 13 centuries.

The partial co-segregation of an FTLD mutation and a genetic risk variant for FTD in this cohort enabled us to compare the clinical features of *GRN*+/A152T+ individuals with those exclusively carrying the *GRN* mutation. Both groups shared similar clinical and neuropsychological profiles. The *GRN*+/A152T+ group showed a trend towards poorer performance in frontal-executive tests and more decline in their MMSE scores at 1 year, but these findings did not reach statistical significance. These results suggest that the p.A152T variant has a limited influence on clinical phenotype in symptomatic c.709-1G>A *GRN* carriers. Serum progranulin levels were not significantly different in the two groups, though *GRN*+/A152T+ tended to have lower progranulin levels.

In contrast, the analysis of brain autopsies suggests that the *MAPT* variant may possibly influence neuropathology. As expected, all autopsy cases showed the FTLD-TDP type A pathology characteristic of *GRN* mutation carriers. Interestingly, variable degrees of tau pathology also emerged: one patient had unusually high tau burden and was designated with an additional diagnosis of an unclassifiable tauopathy, while two patients showed tau pathology accompanied by amyloid deposition and thus received a pathologic diagnosis of Alzheimer’s disease. Previous neuropathological analysis of autopsies of individual patients carrying the p.A152T *MAPT* variant have shown different types of abnormal tau accumulation: NFTs, NTs, tufted astrocytes, and oligodendroglial coiled bodies, in different combinations and distributions. Some fulfilled criteria for corticobasal degeneration, progressive supranuclear palsy [26], or pallidonigroluysian atrophy [27], while others were considered to have unclassifiable tauopathies [28]. A few studies have systematically analyzed the co-emergence of FTLD tau and TDP-43 pathology. Specifically, tau pathology is generally scant and restricted to the hippocampus and/or entorhinal cortex in most patients with FTLD-TDP [29]. Moreover, previous studies suggest that *GRN* brains show reduced tau protein expression, and individuals with FTLD associated with *GRN* mutations have a lower propensity for tau pathology than do other FTLD types, such as FTLD associated with *C9ORF72* mutations or sporadic FTLD-TDP [29,30,31]. Thus, the fact that half of our *GRN*+/A152T+ cases showed significant tau pathology, which appears atypical for *GRN* mutation carriers, may reflect an influence of the p.A152T *MAPT* variant.

We have analyzed the influence of a rare genetic *MAPT* variant on the clinical phenotype of *GRN* carriers. Additional genetic risk factors are not usually assessed in the clinical setting once the disease is explained by a single mutation. However, genetic variants may account for the clinical heterogeneity in these apparently monogenic mendelian diseases, such as autosomal dominant-FTLD caused by *GRN*, *C9ORF72* or *MAPT* mutations. In this context, reports of “double mutations” in FTLD-related genes [32–37] outline the likely influence of more than one gene on disease pathogenesis and phenotype, supporting an oligogenic, rather than monogenic disease model.

Limitations of this study include its relatively small sample size, which poses a challenge for determining the pathogenic nature of the p.A152T *MAPT* variant in our study population and clinical data obtain by retrospective chart review. Second, our results may be unique to families of Basque descent due to their specific genetic background. Nevertheless, we anticipate that future in-depth genetic analyses of families carrying pathogenic mutations will shed light on potential genetic modifiers and disease mechanisms in these complex, heterogeneous neurodegenerative diseases.

## Supporting information

S1 Fig Family 1 pedigree. The symbol * represents subjects that contributed to the haplotype analysis.
(TIFF)

S2 Fig Family 2 pedigree. The symbol * represents subjects that contributed to the haplotype analysis. The symbol + represents the three *GRN*+/*A152T*- subjects from this family cited in the text.
(TIFF)
