# Supplementary figures and images for: The unexpected co-occurrence of GRN and MAPT p.A152T in Basque families: Clinical and pathological characteristics
Source: PLoS One. 2017 Jun 8;12(6):e0178093. doi: 10.1371/journal.pone.0178093 (PMC5464560; doi:10.1371/journal.pone.0178093)

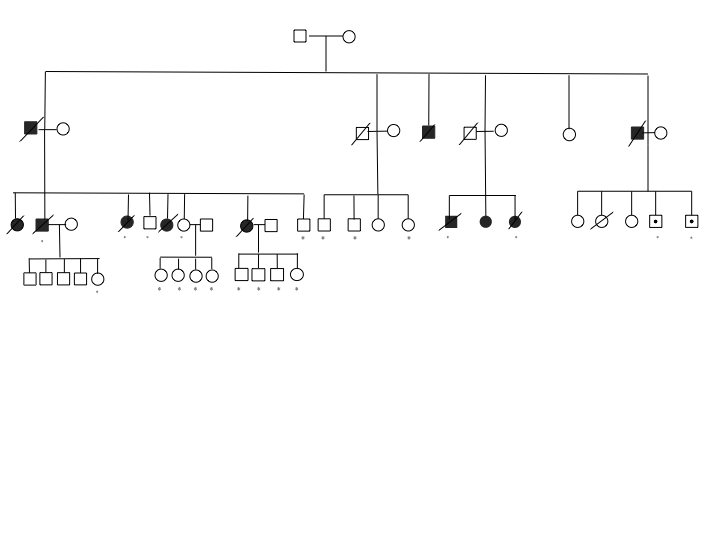

Supplement: S1 Fig — The symbol * represents subjects that contributed to the haplotype analysis. (TIFF) [file pone.0178093.s001.tiff]

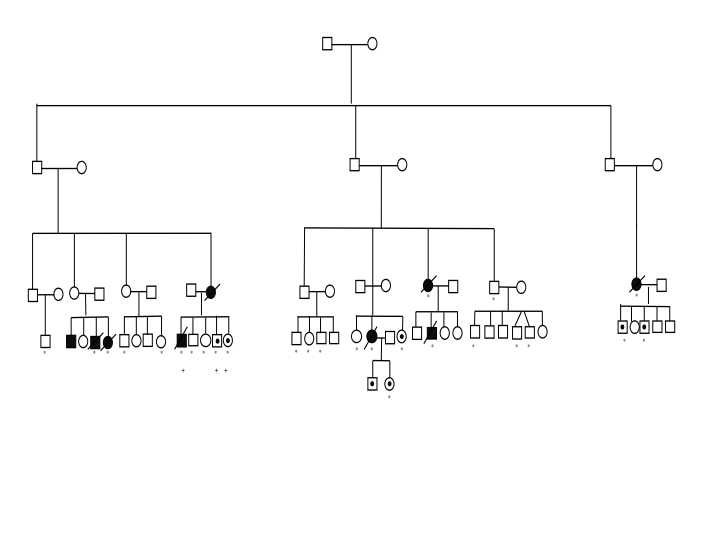

Supplement: S2 Fig — The symbol * represents subjects that contributed to the haplotype analysis. The symbol + represents the three GRN+/A152T- subjects from this family cited in the text. (TIFF) [file pone.0178093.s002.tiff]
